# Disequilibrium, Rather than Postural Orthostatic Tachycardia Syndrome, Is the Primary Determinant of Orthostatic Intolerance in Patients with Long COVID

**DOI:** 10.3390/jcm15062263

**Published:** 2026-03-16

**Authors:** Kunihisa Miwa

**Affiliations:** Department of Internal Medicine, Miwa Naika Clinic, Toyama 930-0002, Japan; info@miwa-naika.com; Tel.: +81-76-482-3014

**Keywords:** long COVID, Myalgic encephalomyelitis, orthostatic intolerance, disequilibrium, postural orthostatic tachycardia syndrome (POTS), standing test

## Abstract

**Background**: Orthostatic intolerance (OI) is an important factor affecting daily functional capacity in patients with long COVID. Traditionally, most OI symptoms have been attributed to exaggerated sympathetic nervous system activation associated with postural orthostatic tachycardia syndrome (POTS). Disequilibrium, also referred to as postural instability, may contribute to the development of OI in patients with long COVID. **Methods**: This study evaluated 32 patients with long COVID using neurological examinations and the active 10-min standing test. Disequilibrium was assessed using the Romberg and tandem gait tests. OI was defined as the inability to complete the active 10-min standing test. **Results**: Seven patients (22%) were diagnosed with OI. None of them had POTS, whereas six (86%) demonstrated disequilibrium, as detected by the Romberg and/or tandem gait test. POTS was observed in eight patients (25%), none of whom had OI. Disequilibrium was observed in nine patients (28%), six of whom (67%) had OI. Multiple regression analysis revealed that disequilibrium was positively associated with OI (r = 0.64, *p* < 0.001), whereas POTS was inversely associated (r = −0.38, *p* < 0.05). After 6 weeks of oral minocycline treatment in six patients and 2 weeks of repetitive transcranial magnetic stimulation therapy following minocycline in the other one patient, symptom amelioration was reported in six patients with OI. OI concomitant with disequilibrium recovered in five of the six patients treated and tested, although one patient who experienced symptom recovery failed to undergo the repeated standing test. **Conclusions**: Disequilibrium, rather than POTS, was the primary determinant of OI in patients with long COVID.

## 1. Introduction

Myalgic encephalomyelitis (ME) [1], formerly known as chronic fatigue syndrome [2], is characterized by persistent malaise or fatigue, cognitive impairment (“brain fog”), unrefreshing sleep, myalgias, arthralgias, post-exertional malaise, and orthostatic intolerance (OI). In recent years, COVID-19 has been reported to cause persistent signs and symptoms, collectively referred to as post-COVID syndrome or long COVID [3], whose clinical presentation closely resembles that of patients with ME [4,5]. Significant overlap exists in the onset, symptom profile, and clinical progression of long COVID and ME [4,5]. OI is an important factor affecting daily functional capacity in patients with ME [6]. Recent reports indicate that many patients with long COVID also experience symptoms related to orthostatic stress, including OI [3,4,5]. OI is characterized by the inability to maintain an upright posture without developing severe signs and symptoms such as palpitations, light-headedness, pallor, fatigue, weakness, dizziness, and nausea [7]. In severe cases, patients with OI may be unable even to sit and may become bedridden. Traditionally, most OI symptoms have been attributed to reduced cardiovascular and cerebral blood flow and to exaggerated sympathetic nervous system activation, often associated with postural orthostatic tachycardia, termed postural orthostatic tachycardia syndrome (POTS) [8,9]. The potential role of disequilibrium in the development of OI has been neglected or ignored. However, recent evidence suggests that disequilibrium, potentially arising from central vestibular dysfunction, may contribute to the pathogenesis of OI in patients with ME [10,11,12,13].

In this study, in order to determine which factor, disequilibrium or POTS, plays a more crucial role in the development of OI in patients with long COVID, disequilibrium was assessed through neurological examinations, and POTS was evaluated during the active 10-min standing test.

## 2. Materials and Methods

### 2.1. Study Population

The study included 32 consecutive patients (15 males and 17 females; mean age, 37 ± 15 years; mean disease duration, 9 ± 7 months) who attended the author’s clinic between April 2020 and February 2025 and met the WHO criteria for long COVID, defined as symptoms persisting for ≥2 months and diagnosed ≥3 months after COVID-19 infection [14]. In this study, only patients who also met the ICC criteria for ME were included [1]. All patients were able to stand and walk. Participants provided written informed consent to participate in the study and the potential publication of identifying information or images in an online open-access format. The study was approved by the Toyama Prefectural Medical Association Ethics Committee (Approval No. 2016-010) and was conducted in accordance with the Declaration of Helsinki.

All patients underwent neurological examinations, including the Romberg and tandem gait tests, to evaluate disequilibrium, as well as a conventional active 10-min standing test. Patients with OI received oral minocycline therapy and/or repetitive transcranial magnetic stimulation (rTMS) and were re-evaluated after treatment.

### 2.2. Neurological Examinations for Disequilibrium

In the Romberg test, patients stood with their feet together and eyes closed for 10 s to assess disequilibrium [13,15]. Unstable standing with wide body sway or oscillations, and any fall with feet together and eyes open or closed, was considered positive for disequilibrium [13,15]. Disequilibrium was also assessed using the 2 m tandem gait test. Patients walked 2 m along a straight, flat surface, making toe-to-heel contact with each step. They then turned around and repeated the tandem gait along the same line [15]. Stepping out, missteps, or body sway accompanied by deviation of the head from the vertical line of gravity at the midpoint between the feet was considered positive for disequilibrium.

### 2.3. Conventional Active 10-Min Standing Test

The conventional active 10-min standing test was performed as previously described [13,15]. Participants stood still with their feet approximately shoulder-width apart. OI was considered present when patients experienced excessive difficulty maintaining the standing posture due to symptoms such as palpitations, light-headedness, pallor, fatigue, dizziness, or nausea, leading them to discontinue standing. POTS was diagnosed when the heart rate increased by ≥30 beats/min in adults and ≥40 beats/min in adolescents under 18 years of age, accompanied by typical symptoms.

### 2.4. Statistical Analysis

Continuous variables are presented as mean ± standard deviation. Student’s *t*-test was used to compare continuous variables. Proportional data were analyzed using Fisher’s exact test. Independent factors including age, sex, disease duration, fibromyalgia, POTS, and disequilibrium, associated with OI were evaluated using a multiple regression analysis (BellCurve for Excel, version 4.07, Social Survey Research Information Co., Ltd., Tokyo, Japan). Statistical significance was set at two-sided *p* < 0.05.

## 3. Results

### 3.1. Detection of POTS

POTS was observed in eight patients, one of whom also exhibited disequilibrium. All eight patients completed the active 10-min standing test.

### 3.2. Detection of Disequilibrium

Disequilibrium was assessed using both the Romberg and tandem gait tests. A positive result was recorded when postural instability markedly worsened during standing, producing wide oscillations and, in some cases, a fall. Seven patients demonstrated a positive Romberg test, and nine patients exhibited a positive tandem gait test. All patients with a positive Romberg test also had a positive tandem gait test. Of the nine patients with disequilibrium, six (67%) failed to complete the active 10-min standing test due to OI. Four of these six had both positive Romberg and tandem gait tests, whereas the remaining two had a positive tandem gait test but a negative Romberg test.

### 3.3. OI with Failure to Complete the Active 10-Min Standing Test

During the active 10-min standing test, seven patients discontinued standing due to symptoms of OI before completing the test, six (86%) of whom had disequilibrium. The average standing duration among these patients was 5.4 ± 1.5 min (Table 1). Comparative data for patients who failed to complete the active 10-min standing test and those who completed it are summarized in Table 2.

Disequilibrium was significantly more prevalent in patients with OI than in those without (*p* < 0.01). Furthermore, comorbidity with fibromyalgia was significantly more prevalent in patients with OI than in those without (*p* = 0.047). Multiple regression analysis, including age, sex, disease duration, presence of fibromyalgia, POTS, and disequilibrium, revealed that only disequilibrium was significantly positively associated with OI (partial regression coefficient: 0.64, *p* < 0.001, 95% confidence interval, 0.32–0.96), whereas POTS was significantly negatively associated (partial regression coefficient: −0.38, *p* = 0.049, 95% confidence interval, −0.31–−0.62). Any other factors were not significantly associated.

### 3.4. Post-Treatment Outcomes

After 6 weeks of oral minocycline therapy [16] in six patients and 2 weeks of rTMS therapy [17] following minocycline in one patient, six patients with disequilibrium reported symptom improvement. OI associated with disequilibrium resolved in five of six treated and retested patients, although one patient experienced symptom recovery without a repeat standing test (Table 1).

**Figure 1:**
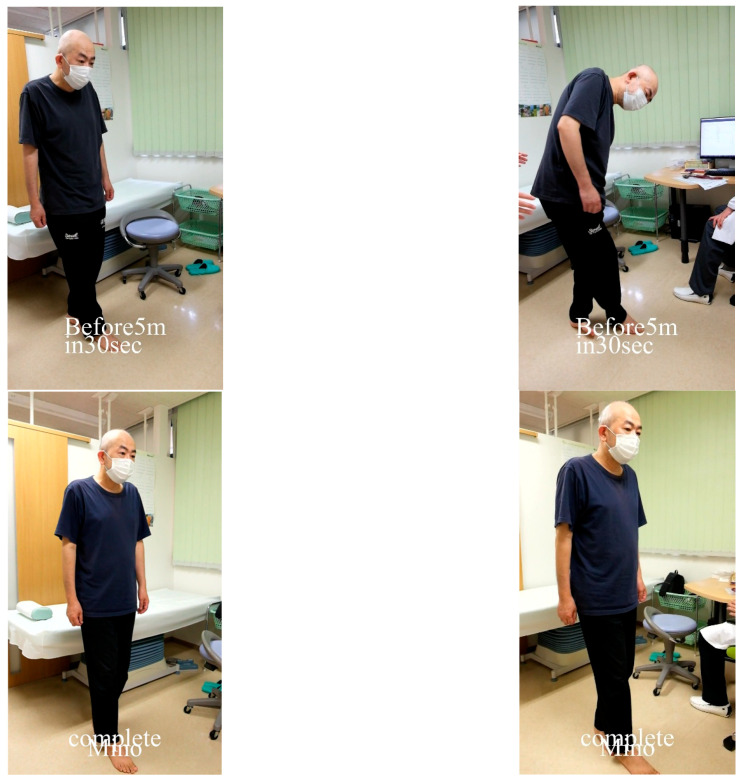
Representative case of a 51-year-old male patient with long COVID for 3 months (Patient #3 in Table 1), who exhibited disequilibrium and was unable to complete the active 10-min standing test. He discontinued standing after 5.5 min due to severe fatigue and light-headedness. Upper panels (left to right): Unstable performance on the tandem gait test, with step-outs, before treatment. Lower panels (left to right): After 6 weeks of oral minocycline therapy, the patient recovered from disequilibrium, demonstrated a stable tandem gait, and completed the active 10-min standing test without significant symptoms.

A representative case is shown in Figure 1.

## 4. Discussion

Almost all researchers have reported that OI is related to POTS associated with reduced cerebral blood flow [7,8,9,18,19,20]. In this study, the conventional active 10-min standing test and neurological examination demonstrated that some patients with long COVID were unable to maintain standing for the full 10 min. Most patients who were unable to complete the active 10-min standing test exhibited disequilibrium, as evidenced by difficulty performing the Romberg test (standing with feet together and eyes closed) and/or the tandem gait test. In addition, a considerable number of patients with disequilibrium failed to complete the active 10-min standing test, whereas nearly all patients without disequilibrium completed the test, except for one, suggesting that disequilibrium or truncal ataxia may play an etiologic role in OI. Notably, none of the patients with POTS failed to complete the test. The presence of disequilibrium appears more important than POTS in the development of OI in patients with long COVID. OI resolved in association with improvement of disequilibrium following treatment with oral minocycline or rTMS. Disequilibrium, combined with postural reflex dysfunction, appears strongly associated with the manifestation of OI.

Postural stability is essential for maintaining static balance, which is necessary for many daily activities. We previously reported that postural instability or disequilibrium, possibly related to central vestibular dysfunction, contributes to the pathogenesis of OI in patients with ME [10,11,12,13].

In a previous study, a review of the records of a patient subgroup at their second visit revealed that some patients who exhibited disequilibrium and were unable to complete the active 10-min standing test had previously completed the test without evidence of disequilibrium, suggesting that OI and disequilibrium developed concurrently during the interim period [12].

Treatment with rTMS has been reported to effectively alleviate various symptoms, particularly OI and disequilibrium, and to improve activities of daily living in patients with ME [17]. The most favorable treatment outcome involved alleviation of disequilibrium, accompanied by resolution of OI [17]. Patients whose disequilibrium improved after rTMS completed the active 10-min standing test, demonstrating resolution of OI and suggesting that postural stability is essential for maintaining an upright posture [17]. In most patients with ME, OI appeared to result primarily from neurological abnormalities in the central nervous system rather than from cardiovascular causes. rTMS likely restored or strengthened neural network function connecting the brainstem vestibular nuclei, cortical vestibular areas, cerebellum, and midbrain, which are redundant, complementary, and overlapping in vestibular function, thereby supporting overall central vestibular function. rTMS should be considered a viable therapeutic option.

A recent pilot trial of minocycline reported favorable effects in 16 of 18 patients with long COVID (89%) [16]. In addition to significant improvement in subjective symptoms including fatigue, post-exertional malaise, unrefreshing sleep, brain fog, and neuropathic pain, recovery from OI, associated with disequilibrium, was observed.

The exact cause of the disequilibrium observed in patients with long COVID remains to be clarified. Although a positive Romberg test suggests a significant visual sensory compensation for the apparent truncal ataxia, proprioceptive sense was not impaired in the patients, suggesting that spinal or sensory ataxia seems unlikely [13]. Also limb ataxia was not observed in the patients, suggesting that the main cause of the ataxia appears to be not of cerebellar origin. It appears to be of central vestibular origin, which is consistent with the previously revealed results of vestibular function tests in patients with chronic fatigue syndrome [21,22], although other mechanisms except central vestibular origin cannot be completely excluded. The vestibular system provides information on head translation, rotation, and orientation in a gravitational environment [23], critically contributing to postural stability. The corticovestibular network among the vestibular nucleus, several vestibular cortex, midbrain, and cerebellum is distributed throughout the brain and has a high degree of functional connectivity [23,24,25]. The pathogenesis of the observed neurologic defect of disequilibrium is probably caused by global neural inflammation in the brain [26].

The head-up tilt test is often employed to diagnose OI and may reveal reduced cerebral blood flow in affected patients [18,19,20]. However, impairments caused by disequilibrium may be masked during head-up tilt testing. Unlike the active standing test, the tilt test where the patient’s body is fully fixed on the tilting board, detects OI resulting solely from circulatory problems, with or without autonomic dysregulation, particularly neurally mediated syncope, but does not detect OI caused by disequilibrium (Table 3). The active standing test appears more suitable than the head-up tilt test for detecting OI in patients with ME or long COVID.

Patients with disequilibrium have been reported to exhibit higher performance status scores indicating greater limitations in activities of daily living than those without disequilibrium, suggesting more severe functional impairment [11,12,13]. Patients with disequilibrium may require greater effort to maintain an upright posture, resulting in exaggerated sympathetic activation and severe fatigue or exhaustion. The negative correlation between POTS and OI observed in patients with long COVID in this study indicates that sympathetic activation associated with POTS serves as a compensatory mechanism necessary for maintaining an upright posture.

This study suggests that neurological testing for disequilibrium including the Romberg, tandem gait, and potentially the single-leg standing tests should be routinely included in the diagnosis of patients with long COVID.

The present study had a couple of limitations. First, the direct evidence showing the causal role of disequilibrium for OI is still lacking. Second, this study had the small sample size of 32 participants with long COVID from a single institute, which may destabilize regression coefficients, inflate correlation estimates, reduce generalizability, and limit subgroup analyses. Obviously, further investigation in a larger number of patients will be required to clarify the precise relation or causal relation between disequilibrium and OI, and its neural origin in patients with long COVID. Also whether disequilibrium is related to possible cerebral hypoperfusion while standing upright in the study patients with long COVID remains to be elucidated.

In conclusion, disequilibrium, rather than POTS, appeared to be the primary determinant of OI in patients with long COVID.

## Figures and Tables

**Table 1:** Clinical characteristics in patients with orthostatic intolerance pre- and after therapy.

Patient	Age/Sex	Disease Duration	Standing Test	Disequilibrium		Therapy	Standing Test *	Disequilibrium *	
#		(Months)		Romberg	Tandem Gait			Romberg	Tandem Gait
**1**	**33/F**	**3**	**7 min dyspnea**	**−**	**+**	**Mino**	**complete**	**−**	**−**
**2**	**30/M**	**8**	**7 min nausea**	**+**	**+**	**Mino, rTMS**	**complete**	**−**	**−**
**3**	**59/M**	**3**	**5.5 min light-headed**	**−**	**+**	**Mino**	**complete**	**−**	**−**
**4**	**36/F**	**9**	**3 min faint**	**+**	**+**	**Mino**	**complete**	**−**	**−**
**5**	**40/F**	**19**	**7 min dizzy**	**+**	**+**	**Mino**	**complete**	**−**	**−**
**6**	**45/F**	**24**	**4.5 min fatigue**	**+**	**+**	**Mino**	**3.8 min nausea**	**+**	**+**
**7**	**34/M**	**20**	**5.4 min malaise**	**−**	**−**	**Mino**	**not done**	**−**	**−**

Orthostatic intolerance: failure to complete the active 10-min standing test, Standing test: duration of maintained standing, Romberg: Romberg test, tandem gait: tandem gait test, +: positive, −: negative, Mino: oral minocycline therapy, rTMS: repetitive transcranial magnetic stimulation, *: post-therapy.

**Table 2:** Clinical characteristics of patients with and without orthostatic intolerance (unable to complete the active 10-min standing test).

	Orthostatic Intolerance		*p*-Value
	(+)	(−)	
**Number of patients**	25	7	
**Male/Female**	12/13	3/4	1.00
**Age (years)**	35 ± 17	41 ± 9	0.36
**Disease Duration (months)**	8 ± 7	12 ± 9	0.21
**Fibromyalgia**	4 (16%)	4 (57%)	0.047
**Postural orthostatic tachycardia**	8 (32%)	0(0%)	0.15
**Disequilibrium**	3 (12%)	6 (86%)	<0.01

**Table 3:** Comparative utilities between the active standing test and head-up tilt test for the diagnosis of orthostatic intolerance (OI).

			Active Standing Test	Head-Up Tilt Test
Conventional			Yes	No
Physiologic			Yes	No
Leg muscle pump function			Available	No
Self-quit			Yes	No
Diagnostic utility				
	Detect OI due to			
		Cardiovascular cause	Yes	Yes
		Autonomic nervous cause	Yes	Yes
				Reflex syncope (++)
		Disequilibrium	Yes	No

++: Head-up tilt tests can more easily induce reflex or neurally mediated syncope.

## Data Availability

Data are available upon request, although availability may be limited due to patient confidentiality. The data associated with this study have not been deposited in any publicly available repository.

## Abbreviations

The following abbreviations are used in this manuscript:

ME	Myalgic encephalomyelitis
CFS	Chronic fatigue syndrome
OI	Orthostatic intolerance
rTMS	Repetitive transcranial magnetic stimulation
